# Effects of Different Ionic Polysaccharides in Cooked Lean Pork Batters on Intestinal Health in Mice

**DOI:** 10.3390/foods11101372

**Published:** 2022-05-10

**Authors:** Xia Yu, Li-Fang Zou, Jia-Hao Xiong, Jing-Zhi Pan, Pei-Jun Li, Cong-Gui Chen

**Affiliations:** 1 School of Food and Biological Engineering, Hefei University of Technology, Hefei 230009, China; 2019010140@mail.hfut.edu.cn (X.Y.); 2019010135@mail.hfut.edu.cn (L.-F.Z.); 2019111373@mail.hfut.edu.cn (J.-H.X.); 2021030069@mail.hfut.edu.cn (J.-Z.P.); lipeijun@hfut.edu.cn (P.-J.L.); 2 Engineering Research Center of Bio-Process, Ministry of Education, Hefei University of Technology, Hefei 230009, China

**Keywords:** polysaccharides, ionic types, low-fat pork batter, intestinal health, intestinal microbiota

## Abstract

The effects of cooked lean pork batters with three ionic types of polysaccharides (anionic xanthan-gum/sodium-alginate, neutral curdlan-gum/konjac-gum and cationic chitosan) on the intestinal health of mice were investigated in this study. The results showed that the zeta potential in the sodium-alginate group (−31.35 mV) was higher (*p* < 0.05) than that in the chitosan group (−26.00 mV), thus promoting the protein hydrolysis in the anionic group because of electrostatic repulsion. The content of total free amino acids in the small intestine in the xanthan-gum and sodium-alginate groups (2754.68 μg and 2733.72 μg, respectively) were higher (*p* < 0.05) than that in the chitosan group (1949.78 μg), which could decrease the amount of undigested protein entering the colon. The two anionic groups could also increase the abundance of *Lactobacillus* and the balance of *Faecalibaculum* and *Alistipes* in the colon. The content of proinflammatory factor IL−6 of colon tissues in the sodium-alginate group (1.02 ng/mL) was lower (*p* < 0.05) than that in chitosan, curdlan-gum and konjac-gum groups (1.29, 1.31 and 1.31 ng/mL, respectively). The result of haematoxylin-eosin staining of the colon also revealed that sodium alginate was beneficial for colonic health. The two neutral groups increased the content of faecal short-chain fatty acids in mice. These results demonstrated that anionic polysaccharides have potential for developing functional low-fat meat products.

## 1. Introduction

Pork meat, as a source of dietary protein, plays an important role in European and Chinese traditional food. However, consumers believe that pork meat contains a high amount of fat with a high content of saturated fatty acids and cholesterol [1], and intake of pork meat proteins also affects colon health by altering the microbiome profile under a complex interaction with adaptive immunity [2]. A high-fat diet is positively correlated with the prevalence and incidence of ulcerative colitis [3]; for example, excessive consumption of saturated fatty acids can increase the prevalence of complex immune-mediated diseases, such as inflammatory bowel disease [4]. Therefore, a healthy eating pattern that includes ample intake of foods rich in fibre and limited in animal fat is recommended [5]. The development of healthy processed meat products with low fat and high fibre is thus a promising field of study.

Polysaccharides are widely used as fat substitutes to improve the gel properties of low-fat meat products [6]. Moreover, the effects of three ionic types of polysaccharides (anionic κ-carrageenan, neutral locust bean gum and cationic chitosan) on the gel properties have revealed that the substitution of anionic and neutral polysaccharides for fat is promising in the development of low-fat meat products [7]. Many reports have also demonstrated that polysaccharides have good physiological functions [8,9]; for example, polysaccharide-induced short-chain fatty acids (SCFAs) and changes in beneficial bacteria (e.g., *Bifidobacterium* and *Lactobacillus*) in the intestinal ecology can have a positive physiological effect on the host [8,10].

A dietary intervention that has substantial effects on human health through modification of the intestinal microbiome may easily provide a powerful approach to disease prevention [11]. In fact, the addition of polysaccharides (resistant starch or inulin) to pork meat products is beneficial for the intestinal health of the host [12,13]. Furthermore, the colloidal status of food components can significantly influence their digestion behaviours and nutritional functions [14]. The stability of emulsions containing proteins and polysaccharides also varies with the ionic type of polysaccharide, resulting in different digestibilities at pH 3–7 [15,16]. Based on the mutual relationship between the intestinal microbiota and its mammalian host, further analysis of the relationship between the gut microbiome and digestion in vivo will help to elucidate the changes in intestinal health when consuming dietary meat containing different ionic types of polysaccharides. However, to the best of our knowledge, no reports have investigated how low-fat cooked meat mixed with different ionic types of polysaccharides influences intestinal health in mice.

In this study, the effects of three ionic types of polysaccharides (anionic xanthan gum/sodium alginate, neutral curdlan gum/konjac gum and cationic chitosan) in cooked lean pork batters on the intestinal health of mice were investigated, in order to provide a theoretical basis for the development of functional meat products with low fat and high fibre.

## 2. Materials and Methods

### 2.1. Materials

Chilled meat from the hindquarters of pork was purchased from the Metro supermarket (Hefei, China). Sodium alginate and chitosan were purchased from Fujifilm Wako Pure Chemical Corporation (Osaka, Japan). Curdlan was provided by MC Food Specialties Inc. (Tokyo, Japan). Konjac gum was supplied by Hubei Yizhi Konjac Biotechnology Co., Ltd. (Yichang, China). Xanthan gum was provided by Deosen Biochemical (Ordos) Ltd. (Zibo, China). Dietary ingredients, including corn starch, dextrinized corn starch, sucrose, soybean oil and cellulose, were purchased from the Metro supermarket. L-cystine, choline bitartrate, mineral mix, vitamin mix and tert-butylhydroquinone were purchased from Sinopharm Chemical Reagent Co., Ltd. (Shanghai, China).

### 2.2. Animals and Diets

The protocol for animal research was approved by the Hefei University of Technology Standing Committee on Animals (HFUT 2019-1111-01). Seventy-two 5-week-old male C57BL/6 mice were provided by Vital River Laboratory Animal Technology Co., Ltd. (Hangzhou, China). The mice were housed in ventilated cages within a pathogen-free barrier facility that maintained a 12 h light–12 h dark cycle and were fed diet ad libitum and allowed free access to water.

The mice were acclimatized after 10 days and were randomly assigned to six experimental diets as follows: (1) dietary excipients containing cooked lean meat powder with free polysaccharide (M); (2) dietary excipients containing a cooked mixture of 0.6% xanthan gum and minced lean meat (XM); (3) dietary excipients containing a cooked mixture of 0.6% sodium alginate and minced lean meat (SM); (4) dietary excipients containing a cooked mixture of 0.6% chitosan and minced lean meat (CSM); (5) dietary excipients containing a cooked mixture of 0.6% curdlan and minced lean meat (CM); and (6) dietary excipients containing a cooked mixture of 0.6% konjac gum and minced lean meat (KM). The feed components of the six diets are shown in Table 1. All formulated diets were prepared according to the recommendations of the American Institution of Nutrition [17]. The casein powder was replaced by meat powder, and polysaccharide was used to replace some cellulose.

Visible fat and connective tissue in lean meat from the hindquarter pork were trimmed off, and the meat was minced twice through a 3 mm plate of a meat grinder at 4–8 °C and then mixed with different polysaccharides (0.6% of minced meat weight) and sodium chloride (1% of meat weight). The meat mixture was stuffed into polyethylene composite nylon bags (approximately 350 g each, 25 × 20 × 6 mm) and cooked for 40 min in an 80 °C water bath. The cooked pork batters were freeze-dried and ground into meat powder. The measurements were completed weekly in the body weight and food intake of the mice. In the final 4 days of the experiment, mouse faeces were collected by grabbing the tail and neck. After 8 weeks, the mice were fasted for more than 12 h and then euthanized with carbon dioxide. Gut tissues, digesta (small intestine and colon), and liver were harvested for the analysis. The mice were randomly allocated to various measurements.

### 2.3. Zeta Potential and pH Value of the Small Intestinal Digesta

According to previously detailed methods [16], the zeta potential of the particles was measured using a Nano-ZS 90 Zetasizer (Malvern Instruments, Worcestershire, UK). The small intestinal digesta were diluted (1:100) using ultrapure water. The values of zeta potential were obtained from three measurements. Additionally, the small intestinal digesta were diluted (1:50) using ultrapure water and the diluted samples were used to measure pH value.

### 2.4. Free Amino Acid Profile of Small Intestinal Digesta

The small intestinal digesta were weighed and dissolved in nine times (*w*/*v*) their volume of normal saline. The specimens were vortexed briefly and centrifuged at 12,000× *g* for 10 min at 4 °C. The supernatant fractions were collected and then treated as described the method previously [18]. The free amino acids were determined using an automatic amino acid analyser (Hitachi-8900, Tokyo, Japan).

### 2.5. Biochemical and Morphologic Analysis of the Colon

The remaining whole colons except for a small amount of tissue used for staining sections, were weighed at 100 mg and ground in nine times (*w*/*v*) their volume of phosphate-buffered solution (pH 7.4). The grinding liquid was centrifuged at 5000× *g* for 15 min at 4 °C, and the supernatant was separated and analysed. The colonic expression levels of interleukin (IL)-6, IL-8, IL-10 and tumour necrosis factor-α (TNF-α) were measured by an enzyme-linked immunosorbent assay (ELISA) with corresponding mouse ELISA kits (Meibiao. Biology Ltd., Yancheng, China).

Freshly isolated mouse distal colons were fixed in 4% paraformaldehyde for histological analysis. The paraffin-embedded tissues were sectioned and stained with haematoxylin and eosin (H&E). The crypt depth, mucosal thickness and thickness of the muscularis (five measurements per slide, three slides per group) were measured. These data were analysed using an Eclipse Ci light microscope (Nikon, Tokyo, Japan) and Image-Pro Plus 6.0 software (Media Cybernetics, Inc., Rockville, MD, USA).

### 2.6. Deoxyribonucleic Acid Isolation, Polymerase Chain Reaction Amplification and Illumina MiSeq Sequencing

The extraction and determination of deoxyribonucleic acid concentration and purity for the colonic contents were performed based on the previous literature [13]. The hypervariable region V3-V4 of the bacterial 16S rRNA gene was amplified with primer pairs 338F (5′-ACTCCTACGGGAGGCAGCAG-3′) and 806R (5′-GGACTACHVGGGTWTCTAAT-3′). Purified amplicons were pooled in equimolar amounts and paired-end sequenced (2 × 300) on an Illumina MiSeq platform (Illumina, San Diego, CA, USA) according to the standard protocols by Majorbio Biopharm Technology Co. Ltd. (Shanghai, China).

### 2.7. Short-Chain Fatty Acids (SCFAs) of the Faeces

The faeces were placed into 10 times their weight to volume of ultrapure water, vortexed for 2 min, and then centrifuged at 10,000× *g* for 30 min at 4 °C. The supernatant was used for determining the SCFAs. Determination of the SCFAs was performed according to previously detailed methods [9,13]. The samples were obtained from a mixture of faeces from mice in the same cage, with three independent replications measured.

### 2.8. Statistical Analysis

Statistical analyses were performed using the Statistical Package for the Social Sciences 25.0 software (IBM Corp., Armonk, NY, USA). Normally distributed data were analysed using one-way analysis of variance with Tukey’s post hoc test. Data that did not conform to a normal distribution were analysed using the nonparametric Kruskal–Wallis test. Data are presented as the mean ± standard error of the mean. A *p* value of <0.05 was considered statistically significant.

## 3. Results and Discussion

### 3.1. Zeta Potential

**Figure 1:**
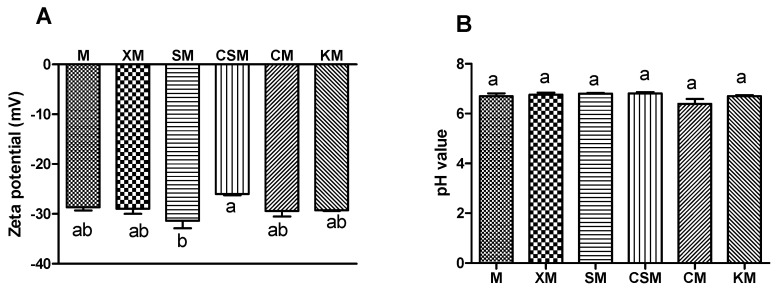
Zeta-potential (**A**) and pH value (**B**) of small intestinal digesta in the mouse. *n* = 12, 12, 12, 11, 12, 12, respectively, for M, XM, SM, CSM, CM and KM. Different letters (a,b) above bars represent statistically significant differences (*p* < 0.05).

Zeta potential is widely used to describe electrostatic interactions between colloidal particles. Its absolute value can be considered as an accumulation of the two zeta-potential values [19]. As shown in Figure 1A, the zeta potential of the SM group was markedly higher than that of the CSM (*p* < 0.05), although there was no statistical significance compared with other groups (*p* > 0.05). Protein generally has a negative charge in the small intestine, where the pH value (Figure 1B) is higher than protein isoelectric point. Moreover, the release of proteins from hydrogel beads is accelerated as the negative charge and electrostatic repulsion increase [20]. These results implied that more proteins were exposed to enzymes and promoted proteolysis in the anionic polysaccharide group with a negative charge due to electrostatic repulsion, compared with CSM.

However, cationic polysaccharides with a positive charge were adsorbed on protein surfaces in the small intestine due to electrostatic attraction, thus blocking the contact between meat proteins and enzymes. Cationic chitosan bind with the negatively charged digestive enzyme and proteins, thereby reducing the activity of digestive enzyme, while anionic alginate benefit for digestive enzyme reaction [15]. Neutral polysaccharides could not contribute to the overall charge characteristics of the systems. The small intestine is the main site for nutrient absorption and is responsible for approximately 90% of the overall energy absorption from the diet [21]. These results implied that the anionic SM group was more conducive to proteolysis than the cationic CSM group and thus had great potential to enhance nutrient absorption.

### 3.2. Free Amino Acids

As shown in Table 2, no significant differences in the total amino acid (TAA) contents were observed among the five dietary groups (*p* > 0.05), except for the cationic CSM group. Compared with the two anionic groups, the CSM group showed a notable decrease in the TAA contents (*p* < 0.05). Compared with the M group, an increase in the contents of cysteine, phenylalanine, tyrosine and arginine was observed in the anionic groups, particularly in the SM group (*p* < 0.05). Furthermore, a remarkable decrease in the contents of asparagine and threonine was observed in the other four groups, but not in the XM group (*p* < 0.05). The free amino acids profiles reflect the hydrolysis degrees of dietary proteins and good physiological function of the host [22]. Arginine, glutamine and leucine play crucial roles in intestinal growth, integrity and function by enhancing mucosal cell migration and restitution [23,24]. Additionally, threonine is using for mucosal and secretory protein synthesis in the intestine [25], and the catabolism of glutamine, glutamate and aspartate provides most of adenosine triphosphate to maintain the intestinal integrity and function [23].

The TAA concentrations in the digesta of cooked lean (low-fat) pork batters with three types of ionic polysaccharides were in order from high to low the anionic polysaccharide group, neutral polysaccharide group and cationic polysaccharide group (Table 2). Both increases in the contents of lysine and arginine were also observed in the anionic groups, compared with the CSM group (*p* < 0.05). Additionally, the specific action sites of digestive enzymes are lysine and arginine [26]. It can be speculated that anionic groups were beneficial to protein release and hydrolysis in comparison with the cationic CSM group. This result may be related to the ionic types of polysaccharides, as they are relevant to the release of protein [20], and this is also in accordance with the zeta potential results (Figure 1A).

In addition, undigested protein and/or unabsorbed amino acids pass into the colon and are subjected to anaerobic fermentation by gut microbes, thus impairing the host’s health [27]. The more protein hydrolysed in the small intestine, the less protein enters the colon for fermentation. High TAA contents in the XM and SM groups (Table 2) imply that more protein is hydrolysed and less undigested protein is fermented, while the cationic CSM group showed the opposite result. These results suggest that the anionic polysaccharide groups are beneficial to the host’s intestinal health compared with the CSM.

### 3.3. Colon Cytokines and Morphology

**Figure 2:**
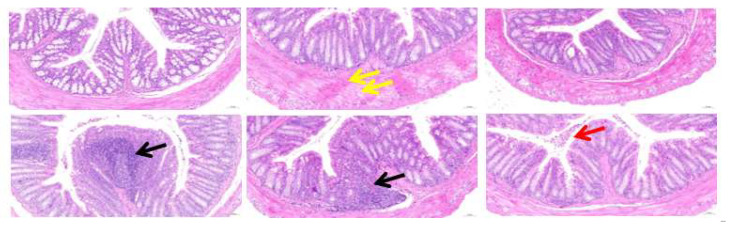
Histomorphology of the mouse colon in response to six dietary groups. Yellow arrow shows swollen muscularis, black arrow shows inflammatory cell infiltration and red arrow shows shedding cells.

As shown in Figure 2, H&E staining of the colon tissues further indicated that the CSM diets were harmful to the colon of mice. The CSM and CM groups showed varying degrees of inflammatory cell infiltration (black arrow), while the KM group revealed an increased number of shedding cells (red arrow) in the colonic lumen of mice. In the XM group, the muscularis was thickened, and the muscle fibres were swollen (yellow arrow). The M and SM groups revealed a normal morphology of mouse colonic tissues.

As shown in Table S1 (Supporting Information), the crypt depth of the CSM group was significantly decreased compared to the M group (*p* < 0.05), and the mucosal thickness of the CSM group was markedly increased compared to the M, XM and CM groups (*p* < 0.05). Although no significant change (*p* > 0.05) in the muscularis thickness was observed in the three types of ionic polysaccharide groups compared to the M group, the muscularis thickness of the XM group was markedly increased compared with the cationic CSM and neutral CM groups (*p* < 0.05). These results indicate that the colonic mucosal barrier was damaged in mice fed the CSM diet.

**Figure 3:**
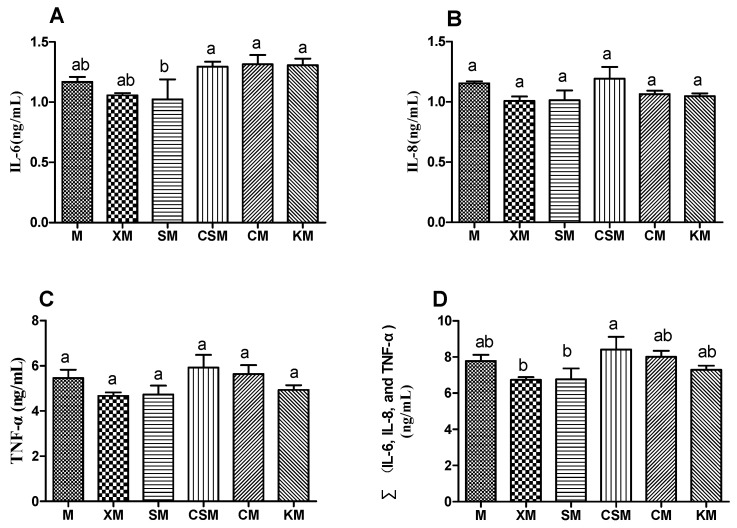
Levels of IL-6 (**A**), IL-8 (**B**), TNF-α (**C**) and ∑(IL-6, IL-8 and TNF-α) (**D**) in the mouse colon in six dietary groups (*n* = 12, 12, 12, 11, 12, 12, respectively, for M, XM, SM, CSM, CM and KM.). Different letters (a,b) above bars represent statistically significant differences (*p* < 0.05).

As described in Figure 3A–C, the anionic SM group demonstrated a significant decrease in IL-6 compared with the cationic CSM and two neutral polysaccharide groups (*p* < 0.05), while no significant difference was observed in the levels of IL-8 and TNF-α in the six groups (*p* > 0.05). Moreover, supplementation with the two anionic polysaccharides may help maintain a healthy intestinal environment by exerting anti-inflammatory effects [28,29]. As described in Figure 3D, the sum of the concentrations of the three proinflammatory cytokines in the anionic XM and SM groups was lower than that in the cationic CSM group (*p* < 0.05). These findings demonstrated that the decreasing of the IL-6 concentration, compared with the CSM, could play a vital role in the anti-inflammatory properties in the anionic polysaccharide groups, especially the SM group.

### 3.4. Microbial Diversity and Composition of the Gut Microbiota

**Figure 4:**
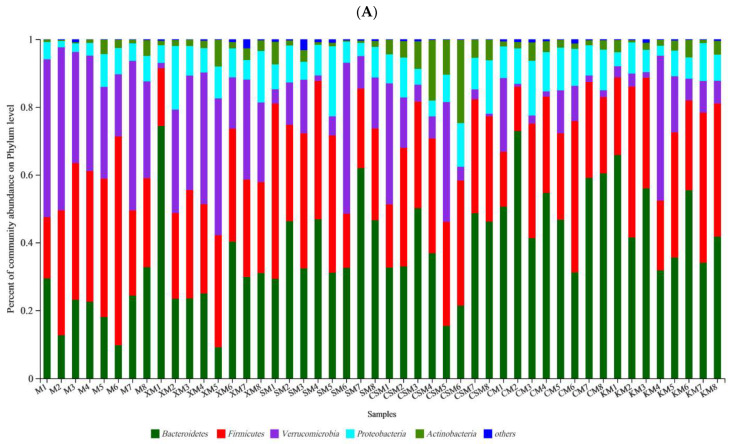

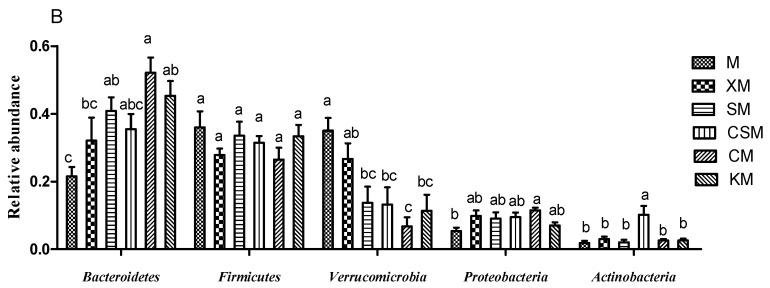
Bacterial taxonomic profiling at the phylum level of gut microbiota (**A**); relative abundance of *Bacteroidetes*, *Firmicutes*, *Verrucomicrobia*, *Proteobacteria* and *Actinobacteria* (**B**); *n* = 8. Different letters (a–c) above bars represent statistically significant differences (*p* < 0.05).

At the phylum level (Figure 4), the three types of ionic polysaccharide diets increased the abundance of *Bacteroidetes* in the mouse intestine, particularly in the two neutral polysaccharide groups and the anionic SM group (*p* < 0.05). The proportion of *Bacteroidetes* in the neutral CM group was significantly higher than that in the anionic XM group (*p* < 0.05). The different polysaccharide diets revealed a significant decrease (*p* < 0.05) in the percent of community abundance of *Verrucomicrobia* except for the XM group. The proportion of *Proteobacteria* in the CM group was increased compared to the M group (*p* < 0.05). The proportion of *Actinobacteria* in the cationic CSM group was markedly higher than that in the other groups (*p* < 0.05). No significant difference was observed in the proportion of *Firmicutes* among the six groups (*p* > 0.05).

**Figure 5:**
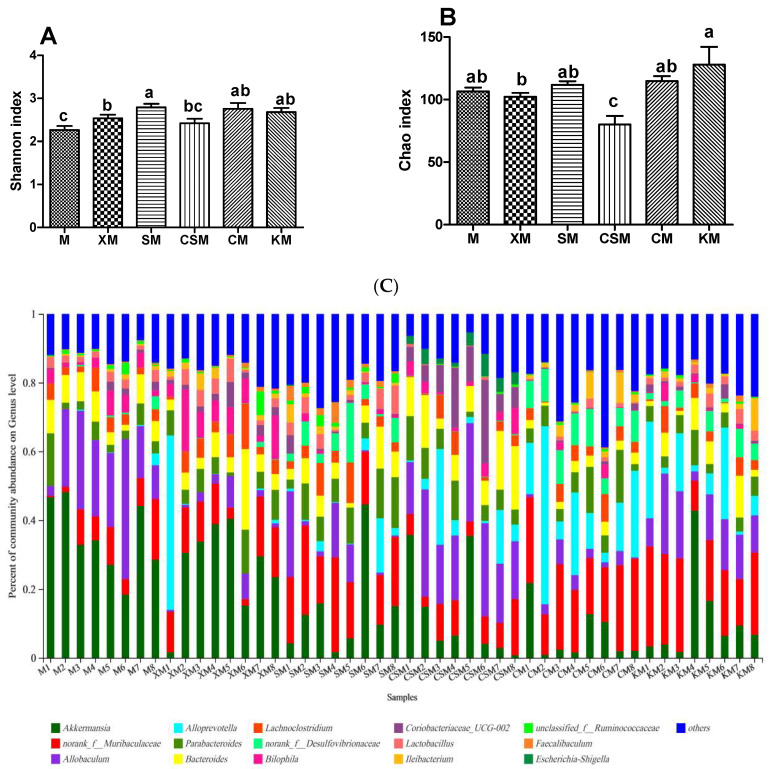

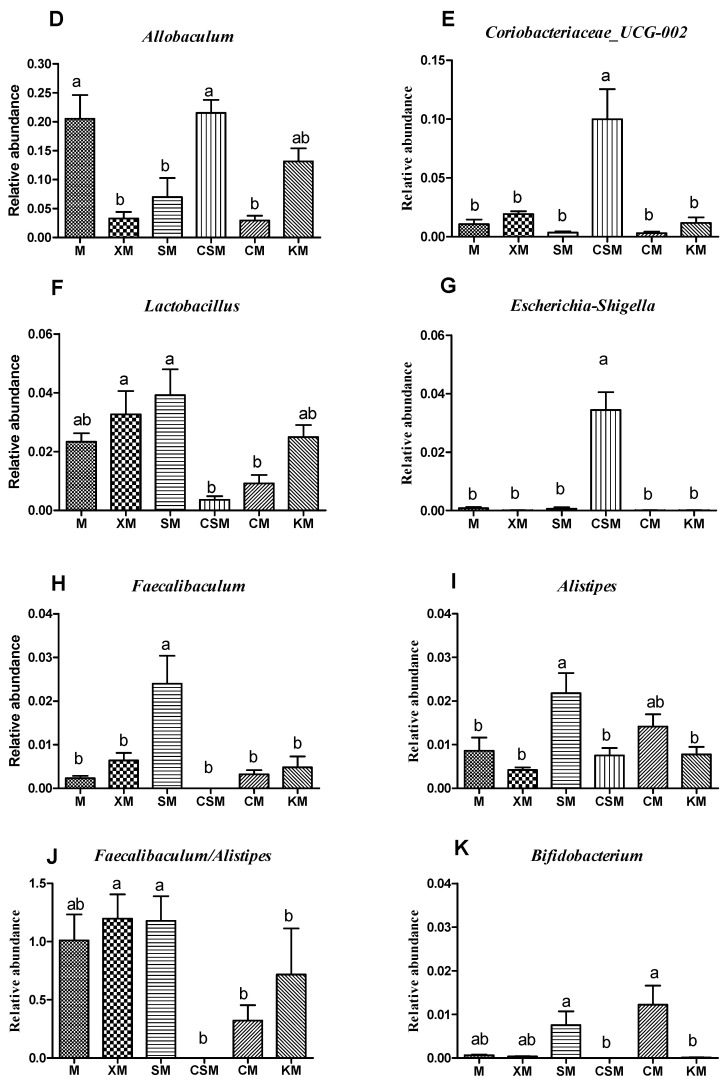
Shannon index (**A**); Chao index (**B**); bacterial taxonomic profiling of gut microbiota (**C**); relative abundance of *Allobaculum* (**D**); *Coriobacteriaceae*_*UCG*-*002* (**E**); *Lactobacillus* (**F**); *Escherichia*-Shigella (**G**); *Faecalibaculum* (**H**); *Alistipes* (**I**); *Faecalibaculum*/*Alistipes* (**J**); and *Bifidobacterium* (**K**). *n* = 8. Different letters (a–c) above bars represent statistically significant differences (*p* < 0.05).

The Shannon and Chao indices were used to appraise the effects of different ionic polysaccharide diets on the diversity and richness of the intestinal microbial community in mice. At the genus level (Figure 5A,B), the diversities of the anionic polysaccharide and neutral polysaccharide groups were higher than that of the M group (*p* < 0.05), and the diversity of the SM group was higher than that of the XM and CSM groups (*p* < 0.05). The richness of the CSM group was significantly lower than that of the other five groups (*p* < 0.05). The richness of the XM group was significantly lower than that of the KM group (*p* < 0.05).

Individuals with low bacterial richness are characterized by increased overall adiposity, a considerably more pronounced inflammatory phenotype than those with high bacterial richness [30]. It has been demonstrated that chitosan derivatives with high numbers of deacetylated units could decrease bacterial populations in vitro [31]. The low richness of cationic CSM (Figure 5B) implies that cationic polysaccharides as fat replacers could increase the risk of intestinal disease in low-fat meat products.

To profile the changes of the gut microbiota, the distribution of bacteria with a relative abundance greater than 0.05 for each dietary group at the genus level is shown in Figure 5C. The levels of the diversity and richness of the intestinal microbial community were low in the CSM group (Figure 5A,B), with *Coriobacteriaceae_UCG-002* and *Escherichia-Shigella* being the dominant microbes (Figure 5E,G). However, both *Faecalibaculum* and *Bifidobacteria* disappeared in the CSM group (Figure 5H,K). It has been reported that sharply increased *Escherichia-Shigella* can exacerbate gut leakiness by penetrating the intestinal epithelial barrier [32]. Bacterial populations of *Escherichia-Shigella* were increased when casein was added to the faecal inoculates, and the bacterial populations of *Bifidobacteria* were decreased when beef protein was added to the faecal inoculates [33]. It is reasonable to infer that the increased populations of both *Escherichia-Shigella* and *Coriobacteriaceae_UCG-002* and the disappearance of both *Bifidobacteria* and *Faecalibaculum* could be attributed to more protein entering the colon for fermentation in the CSM group, leading to a high concentration of the three proinflammatory cytokines (Figure 3D).

*Lactobacillus* has been recognized as having a beneficial impact on gut barrier function [34]. As shown in Figure 5F, *Lactobacillus* is a dominant type of microbe in XM and SM. A significant increase in *Lactobacillus* relative abundance was observed in the anionic XM and SM groups when compared to CSM and CM only. Typically, anionic alginate and its derivatives can be fermented by beneficial bacteria in the large intestine [10,35] and these bacteria can inhibit the formation of putrefactive compounds [36]. Anionic xanthan may help in maintaining a healthy intestinal environment [28]. These results showed that *Lactobacillus* plays a vital role in maintaining colon health in the anionic XM and SM groups.

Interestingly, the relative abundances of *Faecalibaculum* and *Alistipes* in the anionic SM group were markedly higher (*p* < 0.05) than those in the other groups except for the CM group. However, the ratio of *Faecalibaculum* to *Alistipes* showed no significant difference between these two anionic groups, while the ratios of the two anionic groups were higher (*p* < 0.05) than that of the cationic CSM group (Figure 5H–J). *Faecalibaculum* produces SCFAs that contribute to controlling tumour cell proliferation in mice [37]. Although *Alistipes* was reported to be related to colitis [38], the present results suggested that the balance of *Faecalibaculum* and *Alistipes* was correlated with colon health based on the results for the proinflammatory cytokines (Figure 3).

### 3.5. SCFAs of Mice Faeces

Straight-chain SCFAs including acetate, propionate and butyrate are generally considered to possess health benefits [39]. Butyrate can induce an increased thickness of the mucosal layer to maintain the barrier integrity [40], and inhibit proinflammatory cytokines such as IL-6, IL-8 and TNF-α [41]. These straight-chain SCFAs are produced mainly by carbohydrate fermentation, while branched-chain SCFAs (isobutyrate and isovalerate) are generated by the fermentation of protein-derived branched-chain amino acids (BCAAs) such as valine, leucine and isoleucine [39].

**Figure 6:**
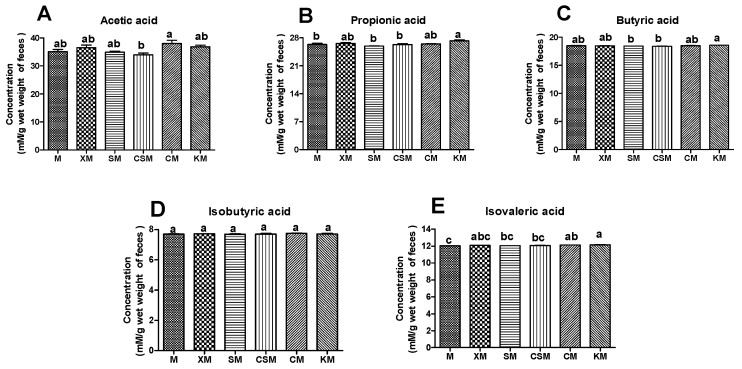
Concentrations of acetic acid (**A**), propionic acid (**B**), butyric acid (**C**), isobutyric acid (**D**) and isovaleric acid (**E**) in the mouse faeces in six dietary groups. The faeces (*n* = 12, 12, 12, 11, 12, 12, respectively, for M, XM, SM, CSM, CM and KM) were from mixture of each cage mouse. Different letters (a,b) above bars represent statistically significant differences (*p* < 0.05).

As shown in Figure 6A–C, the CM group had a significant increase in acetic acid compared with the CSM group (*p* < 0.05). The KM group showed a significant increase in both propionic acid and butyric acid compared to the SM and CSM groups (*p* < 0.05). A high concentration of SCFAs was correlative with a high proportion of SCFA-producing bacteria [10]. The low concentration of butyrate in the CSM group could be ascribed to the low proportion of Lactobacillus (Figure 5F) and the disappearance of *Bifidobacteria* (Figure 5K), thus resulting in unhealthy intestines in CSM-fed mice (Figure 2 and Figure 3). However, the low content of butyric acid in the SM group (Figure 6C) is inconsistent with the high proportion of straight-chain SCFA-producing *Lactobacillus*, *Faecalibaculum* and *Bifidobacteria* (Figure 5F,H,K). Moreover, the healthy intestines of SM-fed mice (Figure 2 and Figure 3) could be related to the contribution of butyrate [41]. It is reasonable to infer that butyrate was produced in abundance in the SM group and was absorbed in the intestine. The high concentration of straight-chain SCFAs in the two neutral groups of CM and KM (Figure 6A–C) could be attributed to the low absorptivity of these SCFAs in the unhealthy intestine (Figure 2 and Figure 3).

An increase in branched SCFAs in faeces means that protein fermentation is intensified [42,43]. As described in Figure 6D,E, no significant change was observed in the content of isobutyric acid among the six groups (*p* > 0.05). However, a remarkable increase (*p* < 0.05) in isovaleric acid occurred in the KM group compared with the SM, CSM and M groups. Moreover, *Prevotellaceae* has been identified as the main bacteria producing BCAAs [44] and branched-chain SCFAs are fermented from BCAAs [39]. The variation trend of the concentration of isovaleric acid (Figure 6E) in the six groups was basically consistent with that of the relative abundance of *Prevotellaceae* (except KM), that is the CM group had a significant increase compared with the M group (*p* < 0.05) and there was no significant difference (*p* > 0.05) among M, XM, SM and CSM groups (Figure S1, Supporting Information). These results implied that the increase in isovalerate could be attributed to fermented BCAAs of abundant *Prevotellaceae* in the colon of KM-fed mice. Although two neutral polysaccharide groups could produce many SCFAs, they could also increase the health risk of the colon because *Prevotellaceae* is associated with intestinal inflammation [45,46].

### 3.6. Growth Performance in Mice

As shown in Table 3, the anionic XM diet promoted body weight gain in mice (*p* < 0.05) when compared with the M and CSM groups, while no significant difference was observed among other groups (*p* > 0.05). Among the six groups, there were no remarkable changes in food intake, liver weight, small intestine weight and colon weight (*p* > 0.05). The anionic XM group showed a significantly increased body weight gain; furthermore, the level of TAA in the anionic XM and SM groups showed a notable increase compared with CSM group, although the TAA increases in the XM and SM groups were not statistically significant (Table 2). These results implied a better digestibility of nutrients in two anionic groups than in cationic CSM group.

## 4. Conclusions

Compared to the CSM, anionic SM and XM diets could promote protein hydrolysis by electrostatic repulsion, increase the amount of TAA in the small intestine, maintain a healthy intestinal environment by reducing the proinflammatory factors and protect intestinal barrier function by increasing the abundance of beneficial bacteria (*Lactobacillus*) and the balance of *Faecalibaculum* and *Alistipes* in the colon. Two kinds of neutral CM and KM could increase microbial community diversity and the contents of branched SCFAs and decrease the absorptivity of straight-chain SCFAs in the intestine, resulting in damage to the colon. A cationic CSM diet could increase the abundance of harmful bacteria (*Escherichia*-*Shigella* and *Coriobacteriaceae*_*UCG*-*002*). These results demonstrated that a cooked low-fat pork batter with anionic polysaccharides, especially SM, was beneficial to intestinal health in mice compared with cationic and neutral polysaccharides.

## Figures and Tables

**Table 1:** Components of six diets with different ionic polysaccharides *.

Ingredient	Diets (g kg^−1^)					
	M	XM	SM	CSM	CM	KM
Meat powder (Corresponding minced meat)	200 (800)	200 (800)	200 (800)	200 (800)	200 (800)	200 (800)
Cellulose	50	45.2	45.2	45.2	45.2	45.2
Xanthan gum	0	4.8	0	0	0	0
Sodium alginate	0	0	4.8	0	0	0
Chitosan	0	0	0	4.8	0	0
Curdlan	0	0	0	0	4.8	0
Konjac gum	0	0	0	0	0	4.8
Sodium chloride	8	8	8	8	8	8
Corn starch	397	397	397	397	397	397
Dextrinized corn starch	132	132	132	132	132	132
Sucrose	100	100	100	100	100	100
Soybean oil	70	70	70	70	70	70
l-Cystine	3	3	3	3	3	3
Choline bitartrate	2.5	2.5	2.5	2.5	2.5	2.5
Mineral mix (AIN-93G-MX)	32.4	32.4	32.4	32.4	32.4	32.4
Vitamin mix (AIN-93G-VX)	10	10	10	10	10	10
Tert-butylhydroquinone	0.014	0.014	0.014	0.014	0.014	0.014
Total	1004.914	1004.914	1004.914	1004.914	1004.914	1004.914

* M: Sodium chloride and minced raw lean pork meat were mixed, cooked, chilled, freeze-dried, ground into powder in sequence, and then other excipients were added to the mixed powder. XM, SM, CSM, CM and KM: Sodium chloride, corresponding polysaccharide and minced raw lean pork meat were mixed, cooked, chilled, freeze-dried, ground into powder in turn, and then other excipients were mixed to the powder. The mineral mix is free of sodium chloride. Each 800 g of minced raw lean meat is the equivalent of 200 g of meat powder.

**Table 2:** Free amino acids of small intestinal digesta in response to six dietary groups *.

Amino Acids	M	XM	SM	CSM	CM	KM
Asparagine	97.39 ± 22.92 ^ab^	112.39 ± 8.40 ^a^	0.00	0.00	0.00	0.00
Threonine	115.08 ± 26.15 ^a^	135.83 ± 7.28 ^a^	74.63 ± 2.71 ^b^	52.69 ± 0.68 ^b^	64.17 ± 9.67 ^b^	64.63 ± 9.17 ^b^
Serine	112.61 ± 21.40 ^a^	124.45 ± 5.00 ^a^	134.32 ± 7.38 ^a^	99.09 ± 1.45 ^a^	122.51 ± 16.78 ^a^	124.72 ± 13.75 ^a^
Glutamine	214.06 ± 49.49 ^a^	255.79 ± 15.25 ^a^	264.53 ± 12.42 ^a^	225.19 ± 9.52 ^a^	230.21 ± 26.05 ^a^	232.84 ± 17.94 ^a^
Glycine	64.65 ± 6.58 ^ab^	70.44 ± 0.81 ^ab^	76.07 ± 5.28 ^a^	55.53 ± 1.06 ^b^	70.90 ± 8.73 ^ab^	72.32 ± 11.27 ^ab^
Alanine	155.70 ± 28.58 ^ab^	179.92 ± 5.46 ^a^	192.47 ± 10.27 ^a^	134.31 ± 2.15 ^b^	172.01 ± 21.38 ^ab^	173.33 ± 11.76 ^ab^
Cysteine	21.83 ± 1.76 ^c^	27.25 ± 0.98 ^ab^	29.62 ± 0.65 ^a^	22.02 ± 0.09 ^c^	24.24 ± 1.99 ^bc^	25.78 ± 1.24 ^b^
Valine	126.04 ± 32.22 ^a^	151.39 ± 7.14 ^a^	160.68 ± 10.14 ^a^	125.15 ± 3.11 ^a^	140.66 ± 19.34 ^a^	144.20 ± 3.77 ^a^
Methionine	78.45 ± 15.93 ^bc^	101.69 ± 3.64 ^a^	96.87 ± 4.64 ^ab^	71.57 ± 2.21 ^c^	90.19 ± 11.26 ^abc^	95.13 ± 1.04 ^ab^
Isoleucine	120.74 ± 33.14 ^a^	141.16 ± 7.97 ^a^	150.40 ± 9.39 ^a^	119.04 ± 1.92 ^a^	131.09 ± 18.55 ^a^	136.35 ± 5.50 ^a^
Leucine	262.10 ± 62.02 ^ab^	323.13 ± 11.81 ^ab^	335.13 ± 18.63 ^a^	241.61 ± 8.42 ^b^	300.82 ± 41.84 ^ab^	306.55 ± 6.35 ^ab^
Tyrosine	172.74 ± 32.25 ^bc^	219.99 ± 7.18 ^a^	222.29 ± 4.95 ^a^	153.67 ± 9.36 ^c^	215.20 ± 20.93 ^ab^	217.78 ± 11.37 ^ab^
Phenylalanine	199.42 ± 37.78 ^bc^	248.31 ± 5.69 ^ab^	259.00 ± 17.80 ^a^	177.77 ± 13.61 ^c^	236.32 ± 23.88 ^ab^	239.20 ± 10.37 ^ab^
Lysine	210.09 ± 30.41 ^ab^	244.48 ± 2.57 ^a^	258.40 ± 11.00 ^a^	181.52 ± 3.77 ^b^	242.37 ± 28.60 ^a^	244.02 ± 14.57 ^a^
Histidine	50.63 ± 6.57 ^ab^	58.46 ± 1.16 ^a^	62.02 ± 3.19 ^a^	45.12 ± 0.54 ^b^	58.18 ± 5.92 ^a^	58.16 ± 5.82 ^a^
Arginine	241.04 ± 43.05 ^bc^	285.51 ± 5.87 ^ab^	305.92 ± 10.81 ^a^	187.32 ± 2.30 ^c^	286.51 ± 34.49 ^ab^	292.61 ± 9.73 ^ab^
Proline	66.08 ± 12.39 ^a^	74.50 ± 5.18 ^a^	76.14 ± 4.72 ^a^	58.19 ± 2.17 ^a^	65.70 ± 11.36 ^a^	67.12 ± 4.99 ^a^
Total amino acid	2308.64 ± 454.91 ^ab^	2754.68 ± 86.63^a^	2733.72 ± 74.80^a^	1949.78 ± 49.29 ^b^	2451.09 ± 299.30 ^ab^	2531.04 ± 103.13 ^ab^

* *n* = 6. Different letters ^(a–c)^ on the same row represent statistically significant differences (*p* < 0.05).

**Table 3:** Growth performance and visceral weight in response to six dietary groups *.

Diet Groups	Body Weight Gain (g)	Food Intake (g/week)	Liver Weight (g)	Small Intestine Weight (g)	Colon Weight (g)
M	2.97 ± 2.09 ^b^	18.88 ± 2.56 ^a^	0.96 ± 0.12 ^a^	0.84 ± 0.21 ^a^	0.23 ± 0.14 ^a^
XM	5.05 ± 2.04 ^a^	22.03 ± 1.33 ^a^	1.02 ± 0.15 ^a^	0.82 ± 0.13 ^a^	0.26 ± 0.14 ^a^
SM	3.34 ± 1.00 ^ab^	20.61 ± 1.49 ^a^	1.00 ± 0.10 ^a^	0.75 ± 0.16 ^a^	0.17 ± 0.10 ^a^
CSM	2.70 ± 1.61 ^b^	22.02 ± 1.57 ^a^	0.97 ± 0.10 ^a^	0.77 ± 0.27 ^a^	0.19 ± 0.04 ^a^
CM	3.52 ± 1.26 ^ab^	22.41 ± 1.99 ^a^	1.02 ± 0.11 ^a^	0.90 ± 0.09 ^a^	0.16 ± 0.03 ^a^
KM	3.36 ± 1.59 ^ab^	20.58 ± 2.33 ^a^	1.03 ± 0.14 ^a^	0.89 ± 0.12 ^a^	0.18 ± 0.04 ^a^

* *n* = 12, 12, 12, 11, 12, 12, respectively, for M, XM, SM, CSM, CM and KM. Different letters in the same column indicate significant differences at *p* < 0.05.

## Data Availability

The datasets generated for this study are available from the authors.

## Supplementary Materials

The following supporting information can be downloaded at: https://www.mdpi.com/article/10.3390/foods11101372/s1, Figure S1: Relative abundance of *Prevotellaceae* in response to six dietary groups; Table S1: Colonic tissue parameters of mice in response to six dietary groups.
