# Supplementary material for: Effects of Different Ionic Polysaccharides in Cooked Lean Pork Batters on Intestinal Health in Mice
Source: Foods. 2022 May 10;11(10):1372. doi: 10.3390/foods11101372 (PMC9141551; doi:10.3390/foods11101372)
Supplement: Supplementary file 1 [file foods-11-01372-s001.zip › foods-1625265-supplementary.pdf]

Supplementary Materials

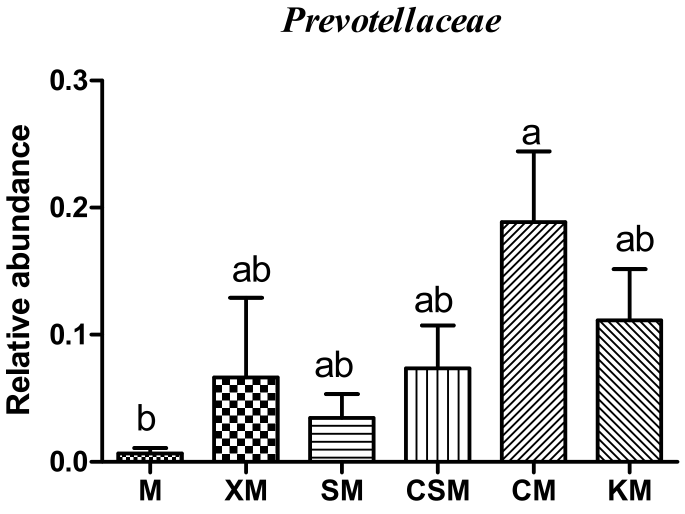

Figure S1. Relative abundance of *Prevotellaceae* in response to six dietary groups *.

* n=8. Different letters (a,b) above bars represent statistically significant differences (p <0.05). M, XM, SM, CSM, CM and KM are as described in **Table 1**.

Table S1. Colonic tissue parameters of mice in response to six dietary groups *

Diet groups	Crypt depth (mm)	Mucousal thickness (mm)	Muscularis thickness (mm)
M	0.180±0.022a	0.232±0.037b	0.114±0.028ab
XM	0.158±0.037ab	0.231±0.025b	0.149±0.029a
SM	0.164±0.042ab	0.239±0.055ab	0.131±0.057ab
CSM	0.146±0.024b	0.274±0.028a	0.095±0.015b
CM	0.157±0.014ab	0.234±0.040b	0.088±0.013b
KM	0.178±0.030ab	0.238±0.038ab	0.137±0.060ab

* n=3, five sections were selected for each tissue section and then were measured. Different letters (a,b) in the same column represent statistically significant differences (p <0.05). M, XM, SM, CSM, CM and KM are as described in **Table 1.**
